# Assessing the Longitudinal outcomes of Piperacillin/tazobactam versus ceftriAxone and metronidazole for Children with perforated Appendicitis (ALPACA): A protocol for a pilot randomized controlled trial

**DOI:** 10.1371/journal.pone.0335991

**Published:** 2025-11-07

**Authors:** Daniel Briatico, Nadia Safa, Helene Flageole, Sarah Khan, Jeffrey Pernica, Mohamed Eltorki, Eyal Cohen, Michael H. Livingston

**Affiliations:** 1 Division of Pediatric Surgery, Department of Surgery, McMaster University, Hamilton, Ontario, Canada; 2 McMaster Pediatric Surgery Research Collaborative, McMaster University, Hamilton, Ontario, Canada; 3 Division of Pediatric Infectious Disease, Department of Pediatrics, McMaster University, Hamilton, Ontario, Canada; 4 Pediatric Emergency Medicine, McMaster Children’s Hospital, Hamilton Health Sciences, Hamilton, Ontario, Canada; 5 Department of Pediatrics, Cumming School of Medicine, University of Calgary, Alberta Children’s Hospital Research Institute, Calgary, Alberta, Canada; 6 Paediatrics and Health Policy, Management and Evaluation, University of Toronto, Toronto, Ontario, Canada; 7 Department of Health Research Methods, Evidence, and Impact, Faculty of Health Sciences, McMaster University, Hamilton, Ontario, Canada; Federal University Oye-Ekiti, NIGERIA

## Abstract

**Background:**

Acute appendicitis is the most common indication for emergency surgery in children. In cases of perforation, patients require post-operative intravenous antibiotics in hospital. However, some children fail to respond adequately, resulting in prolonged hospitalization. The optimal antibiotic regimen for perforated appendicitis remains uncertain.

**Methods:**

We propose a double-blind, randomized controlled pilot trial comparing two commonly used antibiotic strategies. Eligible participants include children <18 years undergoing laparoscopic appendectomy for perforated appendicitis. Following surgery, participants will be randomized to receive either: (1) piperacillin/tazobactam; or (2) ceftriaxone and metronidazole. The sample size for the pilot study is 16 participants (i.e., 8 per group). Feasibility outcomes include recruitment rate, protocol adherence, loss to follow-up, and cost per participant.

**Discussion:**

This pilot study will assess the feasibility of conducting a blinded randomized controlled trial of postoperative antibiotic therapy in children with perforated appendicitis. To date, only one randomized trial has addressed this question, but it was limited by its single-center design, lack of blinding, and susceptibility to ascertainment bias and other methodological concerns. Findings from this pilot will inform the design of a larger, multicenter study with rigorous blinding and standardized outcome assessment to determine whether piperacillin-tazobactam or ceftriaxone and metronidazole provides superior outcomes.

**Trial Registration:**

ClinicalTrials.gov: NCT05943223

## Background

Acute appendicitis is the most common indication for emergency surgery in children [1]. In developed countries, the standard of care includes pre-operative intravenous (IV) antibiotics followed by laparoscopic appendectomy [2]. If the appendix is found to be perforated at the time of surgery, patients must stay in hospital for post-operative IV antibiotics. Patients who do not respond to antibiotic therapy may experience prolonged length of stay, need for additional interventions (e.g., percutaneous drain insertion or parenteral nutrition), or other complications [3]. These outcomes represent significant morbidity for patients and their families.

Historically, children with perforated appendicitis were treated with post-operative ampicillin, gentamicin, and metronidazole (also known as “triple therapy”). In 2008, a randomized controlled trial (RCT) showed that triple therapy was non-inferior to ceftriaxone and metronidazole (CM) in terms of intra-abdominal abscess formation and wound infection [4]. CM is also less expensive and has a simplified dosing regimen. Accordingly, post-operative CM became the standard of care for perforated appendicitis at most children’s hospitals in the United States and Canada [5].

In 2021, an open-label RCT (n = 162) suggested that piperacillin/tazobactam (PT) was more effective than CM for children with perforated appendicitis [6]. Patients randomized to PT had significantly lower rates of intra-abdominal abscess formation (odds ratio [OR] 4.80, p = 0.002), required fewer postoperative imaging studies, and had fewer emergency room visits after discharge [6]. However, the trial had several limitations. It was not blinded, there was no allocation concealment, and the primary outcome—detection of intra-abdominal abscess on postoperative imaging—was subject to potential ascertainment bias. Participants in the CM group underwent imaging more frequently, and the decision to order imaging was left to the treating physician, who was aware of each patient’s antibiotic assignment. This is particularly concerning since postoperative imaging may detect fluid collections that are not clinically significant.

In contrast, a multicenter observational study (n = 654) found no difference between children treated with PT versus CM in intra-abdominal abscess rates (OR 0.88, 95% CI 0.38–2.03, p = 0.77) or resource utilization, including post-operative imaging, length of stay, and hospital cost [7]. Likewise, another study (n = 1,002) reported comparable rates of organ space infection (17.0% vs 13.1%, p = 0.08), post-operative drainage (11.7% vs 10.7%, p = 0.63), and post-operative length of stay (median 4 vs 4 days, p = 0.26) between groups [8]. Interestingly, the largest multicenter observational study (n = 29,015) found PT to be associated with higher risks of abscess formation (relative risk [RR] 1.35, 99% CI 1.04–1.75) and readmission to hospital (RR 1.38, 99% CI 1.13–1.68) [9]. However, after adjusting for hospitals with high CM utilization (≥75% cases), these differences were no longer statistically significant [9]. Together, these inconsistent results highlight ongoing uncertainty about the optimal antibiotic regimen. Furthermore, all studies were conducted in the United States [6–9], limiting generalizability to Canadian patients who may have different microbial sensitivities and antibiotic resistance patterns [10,11].

Another important consideration is antibiotic stewardship [12]. PT is a broad-spectrum antibiotic with increased effectiveness against *Pseudomonas aeruginosa* and resistant strains of *Escherichia coli*. However, the overuse of broad-spectrum antibiotics can promote the emergence of drug-resistant pathogens, which are associated with worse clinical outcomes [13]. Compared with narrow-spectrum agents, broad-spectrum antibiotics are also linked to a higher risk of adverse effects, risks that are often underappreciated by healthcare providers and the general public [14]. Using PT for a routine, community-acquired infection like perforated appendicitis may result in unnecessary exposure to broad-spectrum antibiotics, which appears to be associated with long-term harms [15–17].

At McMaster Children’s Hospital, we reported substantial variation in the type of post-operative antibiotics used among children with perforated appendicitis (n = 71) [18]. In this study, we found that some patients received CM alone (n = 32), others received PT (n = 23), and a subset treated initially with CM were “escalated” to PT when their symptoms did not improve (n = 15). Compared to patients treated with CM only (n = 32), those escalated to PT (n = 15) had worse intra-operative findings at baseline, and also showed an increased need for post-operative ultrasound (67% vs 34%, p = 0.038), parenteral nutrition (33% vs 3%, p = 0.004), peripherally inserted central catheter (PICC) insertion (47% vs 3%, p < 0.001), and greater length of stay (10.7 vs 5.1 days, p < 0.001). There were 3 cases of *Clostridioides difficile* infection (4%), but all of these occurred in patients treated with CM.

In short, there remains clinical equipoise regarding whether PT or CM is the better option for children with perforated appendicitis. A single-center, unblinded RCT with some risk of bias suggested a benefit with PT [6], while three multicenter, observational studies reported no difference [7–9]. Given the potential harm and unnecessary exposure to broad-spectrum antibiotics, a well-designed RCT is needed to resolve this uncertainty.

## Methods

### Study design

The Assessing Longitudinal outcomes of Piperacillin/tazobactam versus ceftriAxone and metronidazole for Children with perforated Appendicitis (ALPACA) trial is a pilot study for a multicenter, blinded RCT with two parallel groups. The primary aim of this study is feasibility, not efficacy. Specifically, this pilot will evaluate recruitment, consent, protocol adherence, outcome assessment, and study costs to determine whether a larger multicenter trial can be successfully conducted. The research question for the multicenter trial is: “Among children less than 18 years of age who undergo laparoscopic appendectomy for perforated appendicitis, is post-operative PT superior to CM in terms of length of stay in hospital?” Although we hypothesize that PT may be associated with a shorter length of stay and improvements in other clinically important outcomes, this pilot study is not powered to evaluate efficacy and will not conduct hypothesis testing on clinical endpoints.

### Setting

The pilot study will be conducted at McMaster Children’s Hospital, a tertiary children’s hospital located in Hamilton, Ontario, Canada. Our center performs approximately 250 laparoscopic appendectomies per year, including 1–2 cases of perforated appendicitis each week. This study was approved by the Hamilton Integrated Research Ethics Board (HiREB: 16639; May 2024) and Health Canada (HC: 284846; April 2024). The ethics approved protocol can be found in **S1 Appendix**.

### Usual practice

At McMaster Children’s Hospital, all laparoscopic appendectomies in children are performed by pediatric general surgeons with fellowship training in Pediatric Surgery. Patients found to have perforated appendicitis are treated with PT or CM in hospital post-operatively. Pain is controlled with oral acetaminophen, IV ketorolac (or oral ibuprofen), and IV morphine as needed. Maintenance IV fluids are gradually weaned as an oral diet is introduced and bowel function normalizes.

Patients who are unable to tolerate an oral diet for 7 days (including both pre- and post-operative days) are often considered for PICC insertion and parenteral nutrition. However, decisions about the timing of PICC insertion and initiating parenteral nutrition are ultimately left to the attending surgeon. At our institution, the duration of IV antibiotics is based on clinical criteria. These include: (1) resolution of pain and localized tenderness; (2) resolution of fevers (i.e., any documented temperature greater than 38.0 degrees); (3) resolution of diarrhea; and (4) ability to tolerate an oral diet. If symptoms resolve within one week of surgery, patients are discharged home with a course of oral antibiotics, typically amoxicillin and clavulanic acid.

Patients with persistent symptoms up to one week after surgery typically undergo an abdominal ultrasound to assess for the presence of a phlegmon (i.e., inflamed soft tissue) or drainable abscess (i.e., organized collection of purulent fluid). Patients with a large abscess (i.e., typically greater than 5 cm in maximal dimension) are often considered for percutaneous drain insertion by a pediatric interventional radiologist. In children, this procedure requires a general anesthetic and the drain is left in place for a few days to allow the abscess to evacuate completely. These patients remain in hospital on IV antibiotics until the drain is removed and symptoms resolve.

When patients are discharged home, they are advised to contact our office or return to the emergency department if they have fevers, persistent diarrhea (which may be a sign of an intra-abdominal abscess or *Clostridioides difficile* infection), abdominal pain, or inability to tolerate an oral diet. Some of the patients who return to the emergency department require readmission to hospital for additional IV antibiotics and possible percutaneous drain insertion.

### Participants

Participants in this pilot study will include children treated with laparoscopic appendectomy for perforated appendicitis. The inclusion and exclusion criteria are listed below.

*Inclusion criteria:*

- Age less than 18 years
- Laparoscopic appendectomy
- Perforated appendicitis confirmed intra-operatively (i.e., visible hole in appendix, fecalith found in peritoneal cavity, intra-abdominal abscess, and/or purulent fluid in the peritoneal cavity)

*Exclusion criteria:*

- Non-operative treatment (since patients managed non-operatively have a different clinical course and outcomes compared to those undergoing immediate appendectomy)
- Interval laparoscopic appendectomy (since patients undergo interval laparoscopic appendectomy as a day procedure and do not receive post-operative antibiotics)
- Conversion to open procedure (since this is rare in children undergoing laparoscopic appendectomy, and those requiring open surgery tend to experience prolonged stay in hospital due to more severe disease at baseline, greater need for post-operative pain management, slower return of gastrointestinal function, etc.)
- Non-perforated appendicitis (since children with non-perforated appendicitis do not require post-operative antibiotics)
- Confirmed or suspected allergy to penicillins or cephalosporins (since allergic reactions would compromise safety and necessitate switching post-operative antibiotics to non-study alternatives)
- Renal impairment (since PT may be associated with increased odds of acute kidney injury in critically ill children [19]. Moreover, renal dysfunction alters drug clearance, potentially leading to toxicity or adverse drug reactions [20])
- Weight less than 10 kilograms (since small children have unique physiological and clinical conditions [21]. Antibiotic dosing protocols may not be generalizable to the broader pediatric population)

### Interventions

Participants will be randomized to receive one of the following post-operative antibiotic regimens:

1. Piperacillin/tazobactam 100 mg/kg IV q8h (to a maximum of 4.5 g IV q8h) and normal saline (i.e., placebo) once daily.
2. Ceftriaxone 50 mg/kg IV once daily (to a maximum of 2 g IV daily) and metronidazole 10 mg/kg IV q8h (to a maximum of 500 mg IV q8h).

To maintain blinding, all study participants will receive one study treatment every 8 hours (either PT or metronidazole) and one study treatment every 24 hours (either ceftriaxone or normal saline).

### Outcomes

#### Feasibility outcomes.

The feasibility outcomes for the pilot study include:

- Recruitment rate (i.e., mean number of participants randomized per month)
- Consent rate (i.e., number of participants who consent to participate divided by those who are approached for consent)
- Rate of protocol violations (i.e., number of participants who do not receive study treatments within 8 hours of surgery, miss a scheduled study treatment, and/or experience treatment crossover divided by those randomized)
- Rate of loss to follow-up (i.e., number of participants who cannot be contacted by phone 3 months after discharge from hospital divided by those randomized)
- Study costs per participant randomized (i.e., total cost of pilot study in Canadian dollars divided by the number of participants randomized)

Our criteria for feasibility are recruitment rate ≥1 new participant per month, consent rate >30%, rate of protocol violations <20%, rate of loss to follow-up <20%, and study costs <3000 Canadian dollars per participant randomized. A similar RCT of children with appendicitis reported a consent rate of 50% [22]. This increased from 38% to 72% throughout the course of the study with focused training of clinical and research personnel.

Progression criteria will serve as key benchmarks to evaluate the feasibility of conducting a larger RCT. While each criterion is important, we recognize that feasibility is a multifaceted assessment and we will not base the decision to proceed on any single factor.

#### Primary clinical outcome.

The primary outcome for the multicenter RCT is length of stay in hospital. This is because patients who respond to post-operative antibiotics typically remain in hospital for 5 days or less. Patients who experience prolonged stays in hospital often do so because of persistent infectious symptoms. These include fever, abdominal pain, diarrhea, and/or poor oral intake. The treatment of this subset of patients includes prolonged IV antibiotics, post-operative imaging, possible percutaneous drain insertion, PICC line insertion, and/or parenteral nutrition. This often results in a length of one to two weeks (or longer).

#### Secondary clinical outcomes.

Another goal of the pilot study is to ensure that we can reliably assess secondary outcomes. These are likely to be affected by the type of antibiotic therapy and have been used in other trials of children with perforated appendicitis. The secondary outcomes include:

- Post-operative imaging (i.e., ultrasound or computed tomography)
- Deep or organ-space SSI (i.e., intra-abdominal abscess formation) [23]
- Percutaneous drain insertion
- *Clostridioides difficile* infection (confirmed with stool sample and requiring treatment)
- Parenteral nutrition
- PICC line insertion
- Return to the emergency department within 30 days of surgery
- Readmission to hospital within 30 days of surgery

#### Patient-reported outcomes.

The Research Coordinator for this study will phone participant family members 3 months after discharge to ensure that they have not experienced any additional complications related to perforated appendicitis. Family members will also complete a questionnaire by phone regarding patient satisfaction and rank the relative importance of the primary and secondary outcomes.

### Sample size for pilot study

The sample size of the pilot study will be 16 participants (i.e., 8 per treatment arm). This number is approximately 10% of the total sample size for the multicenter RCT and should be sufficient to assess feasibility and cost [24–26].

### Sample size for multicenter trial

The sample size for the multicenter RCT is 180 participants (i.e., 90 per treatment arm). In our recent quality assurance project, the average length of stay for participants treated with PT only (n = 23) was 5.9 days (standard deviation 2.9 days), compared to 6.9 days (standard deviation 4.2 days) for those who received CM first (n = 32) [18]. The standard deviation for the entire sample (n = 71) was 3.8 days [18].

A parallel two-group design will be used to test whether the PT location (distribution center) is different from the CM location (H0: μ1 - μ2 = 0 versus H1: μ1 - μ2 ≠ 0). The comparison will be made using a two-sided, two-sample Mann-Whitney U test, with a Type I error rate (α) of 0.05. The common standard deviation for both groups is assumed to be 3.8, and the underlying data distribution is assumed to be Normal. To detect a difference in means of 2 with 90% power, the number of needed subjects will be 81 in Group 1 and 81 in Group 2. The sample size was computed using PASS 2024, version 24.0.5. Assuming a dropout rate of approximately 10%, we plan to increase the total sample size to 180 participants.

### Recruitment

Participants for the ALPACA trial will be identified by the attending pediatric surgeon or pediatric surgical fellow. After consent is obtained for surgery (i.e., “laparoscopic possibly open appendectomy”), the patient and family will be asked if they are agreeable to learn more about the study. If so, they will be approached by a member of the research team for possible study enrollment. Informed consent will be obtained from the parent or legal guardian. Participants who are not consented before surgery will not be enrolled. Assent will be obtained from the patient whenever possible.

Patients who decline to participate will receive usual clinical care. This includes pre-operative IV antibiotics followed by laparoscopic appendectomy. If the appendix is not perforated during surgery, the patient will be discharged home. Conversely, if the appendix is found to be perforated, patients will receive PT or CM post-operatively (with the type of antibiotics left to the discretion of the attending pediatric surgeon).

Patients and families who consent to participate in the ALPACA trial will also proceed with surgery as per usual clinical care. If the appendix is found to be perforated at the time of surgery, they will then be randomized to receive PT or CM post-operatively. If the appendix is not perforated, the patient will not be randomized or receive any study treatments. They will return to usual clinical care, which typically involves being discharged home with no further oral antibiotics.

### Randomization

Participants will be randomized to treatment groups using a computer-generated randomization list. This will consist of random blocks of multiple sizes ranging from 2 to 6 individuals, created by a biostatistician. Participants will be randomized in a 1:1 parallel design, with an equal chance of being allocated PT or CM.

### Concealment mechanism

The randomization scheme will be housed in Research Electronic Data Capture (REDCap), a secure web-based application [27]. Participants will be randomized by the research pharmacy at McMaster Children’s Hospital once they have been enrolled in the study, assuring allocation concealment from study investigators and the clinical team. The inpatient pharmacist will possess a study key to determine which study arm the patient is assigned to. The assigned treatments will be crosschecked against the master linkage key at the end of the study.

### Blinding

All participants, caregivers, clinical staff, and outcome assessors will remain blinded throughout the trial. The only reason for emergency unblinding is if a participant develops signs of a moderate to severe allergic reaction (i.e., hives, anaphylaxis, etc.). This will allow the clinical team to determine if the reaction was due to a penicillin (i.e., piperacillin) or cephalosporin (i.e., ceftriaxone). Allergic reactions to IV metronidazole are rare. In addition to unblinding, the participant’s IV antibiotics will be changed to an alternative regimen. At our center, the usual alternative regimen for children with allergies is ciprofloxacin and metronidazole, but the decision will be left to the attending surgeon.

### Follow-up

There should be no loss to follow-up while study participants remain in hospital. After discharge, two National Surgical Quality Improvement Program (NSQIP) data abstractors routinely review the electronic medical record of all children who undergo laparoscopic appendectomy for perforated appendicitis. There is a possibility that participants could develop a complication and present to the emergency department at a different hospital. To capture this, our Research Coordinator will call patient families 3 months after discharge from our center.

### Data collection

The Research Coordinator will be responsible for storing signed consent forms and entering baseline data. Feasibility outcomes will also be assessed by the Research Coordinator. The NSQIP data abstractors will be responsible for assessing and recording the primary and secondary outcomes.

### Data management

All study data will be stored in a REDCap database [27], designed specifically for the purposes of this trial. Data entered into REDCap will be de-identified, with participant identifiers replaced with unique study identification numbers. Any electronic files containing patient identifiers, including the master list linking medical record numbers (MRNs) to study identification numbers, will be password-protected and stored on the secure drive for the Department of Surgery at McMaster University. This drive is protected by the McMaster University firewall, and only research staff directly involved in the conduct of this trial will have access to these files. Paper consent forms will be kept in a locked cabinet within the secure office space for the Department of Surgery. Only research staff directly involved in the conduct of this trial will have access to this cabinet.

To ensure data accuracy, data completeness checks will be conducted throughout the trial. The first check will occur after five participants are randomized, with an additional check upon completion of the pilot study.

### Statistical analysis

The purpose of this pilot study is to assess the feasibility of conducting a full-scale RCT. Accordingly, no hypothesis testing will be performed on any of the primary or secondary clinical outcomes, as the study is not powered to detect statistically significant differences. All analyses will be descriptive.

Feasibility outcomes (which include recruitment rate, consent rate, frequency of protocol violations, loss to follow-up, and cost per participant) will be summarized using descriptive statistics. The primary clinical outcome, length of hospital stay, will be described using measures of central tendency and dispersion (mean, median, standard deviation, and range). Secondary outcomes will be summarized as counts and proportions. Patient-reported outcomes (e.g., satisfaction and outcome rankings) will be summarized descriptively using frequencies and proportions.

Cost per participant randomized will be estimated in Canadian dollars. This estimate will include personnel costs associated with consent, randomization, outcome assessment, and pharmacy services. These data will be used to estimate the budget required for recruitment and implementation of the full-scale trial.

All statistical analyses will be performed using SPSS (IBM Corp., Armonk, NY, USA). A summary of each outcome and corresponding summary statistics can be found in **Table 1**.

**Table 1:** Outcomes, assessments, and summary statistics.

Outcome	Assessment	Summary Statistics
***Feasibility Outcomes***		
Recruitment rate	Number of participants randomized divided by study period	Mean number of participants per month
Consent rate	Number of participants consented per number approached	Count, proportion
Protocol violations	Number of violations per participants randomized	Count, proportion
Loss to follow-up	Number of participants unable to contact at 3-month phone call out of total participants randomized	Count, proportion
Study cost per patient	Study costs divided by total participants randomized	Mean cost per participant randomized
***Primary Clinical Outcome***		
Length of stay	Length of hospital stay during index admission (days)	Mean, median, standard deviation, range
***Secondary Clinical Outcomes***		
Post-operative imaging	Post-operative ultrasound or CT scan	Count, proportion
Deep/organ-space SSI	Post-operative deep/organ-space SSI, per NHSN criteria [23]	Count, proportion
Drain insertion	Percutaneous drain insertion or aspiration by Interventional Radiology	Count, proportion
Clostridioides difficile infection	Infection confirmed by positive stool sample	Count, proportion
Parenteral nutrition	Post-operative parenteral nutrition	Count, proportion
PICC line insertion	Post-operative PICC line insertion	Count, proportion
Return to emergency department	Return to emergency department within 30 days post-surgery	Count, proportion
Readmission to hospital	Readmission to hospital within 30 days post-surgery	Count, proportion
***Patient-Reported Outcomes***		
Patient-reported satisfaction	Survey completed 3 months post-discharge (5-point Likert scale)	Count, proportion

### Data monitoring

This study will have a Steering Committee. The Steering Committee members will be responsible for overseeing the conduct of the trial. The Steering Committee will meet monthly and will include the study co-investigators from Pediatric Surgery, Pediatric Infectious Disease, and Pediatric Emergency Medicine.

### Serious adverse events

SAE reporting will adhere to the guidelines set out by HiREB, which requires reporting of all SAE that are unexpected and related to study treatments (or possibly related). If an unexpected SAE occurs, the Principal Investigator will notify the Data Safety Monitoring Board (DSMB) within 48 hours. The only SAE that will be reviewed by the DSMB are allergic reactions possibly related to study medications. The DSMB will also review any cases of mortality within 30 days of surgery. All unexpected SAEs associated with the drug but are not fatal or life-threatening will be reported to Health Canada within 15 calendar days. Any unexpected SAEs associated with a drug that are fatal or life-threatening will be reported to Health Canada within 7 calendar days, followed by a complete written report within 15 calendar days.

### Data Safety Monitoring Board

This study will have a DSMB made up of three healthcare professionals, independent of the Steering Committee, who will monitor patient safety throughout the study. Their role is to review all SAEs that are unexpected and determine if they are related or possibly related to study treatments. The DSMB will submit a summary report to the Steering Committee and HiREB. Based on these reports, the DSMB will recommend either continuing or discontinuing the trial due to harm.

### Timeline

**Fig 1:**
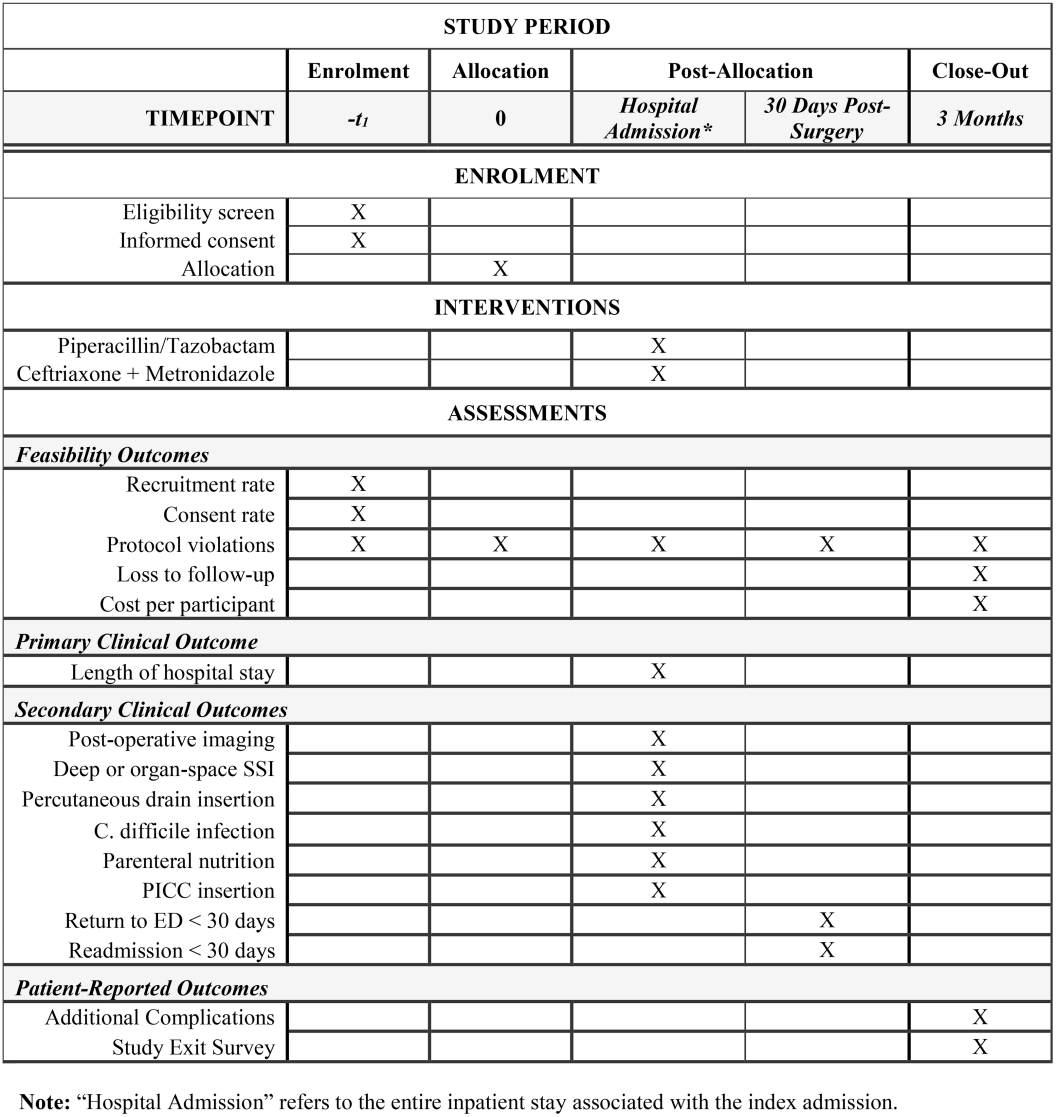
SPIRIT diagram outlining the schedule of enrolment, interventions, and assessments for the ALPACA trial.

At McMaster Children’s Hospital, approximately 60 pediatric patients undergo laparoscopic appendectomy for perforated appendicitis each year. As such, it is estimated that recruitment for the pilot study will require at least one year to complete enrollment. This is based on an estimated recruitment rate of 2–3 participants randomized per month and an estimated consent rate of 50% [22]. Recruitment began on September 9, 2024. A detailed schedule of enrolment, interventions, and assessments for the ALPACA trial can be found in **Fig 1**. The Standard Protocol Items: Recommendations for Interventional Trials (SPIRIT) checklist can be found in **S2 Appendix**.

### Dissemination

The results of the pilot study will be published in a peer-reviewed journal and presented at relevant academic meetings. These include the annual meeting for the Canadian Association of Pediatric Surgeons and the Canadian Pediatric Society. To improve the quality of reporting, findings will be presented in accordance with the CONSORT 2010 extended statement for randomized pilot and feasibly trials [28].

### Confidentiality

Patient medical information will be kept strictly confidential and handled in accordance with Good Clinical Practice (GCP) and the Personal Health Information Protection Act (PHIPA). Only personal identifiers essential for the success of this study will be collected. All data will be anonymized for validation and analysis purposes. Access to de-identified data or the participant master list will be restricted to research staff directly involved in the study.

### Ancillary and post-trial care

All trial data will be kept for 15 years in compliance with Health Canada guidelines. Interested participants and their families can be advised of the results of the pilot study by email.

## Discussion

Perforated appendicitis remains a common cause of morbidity among otherwise healthy children [3]. To date, the optimal antibiotic regimen following laparoscopic appendectomy remains uncertain. While one non-blinded RCT suggested improved outcomes with PT [6], three observational studies found no difference [7–9]. These conflicting findings highlight the need for a rigorously designed RCT. Importantly, the prior RCT lacked blinding and allocation concealment, raising concerns about potential bias and limiting the reliability of its conclusions [6].

RCTs are considered the highest level of evidence [29], but their strength depends on methodological rigor, including adequate blinding, allocation concealment, and unbiased outcome assessment [30]. Despite their importance, less than 2% of the pediatric surgical literature is derived from RCTs [31,32], leaving even common conditions such as appendicitis without robust, high-level evidence.

Given the complexity of conducting surgical trials in pediatric populations [33,34], a feasibility study is an essential first step. Challenges include timely identification and enrollment of patients before emergency surgery, obtaining informed consent in urgent settings, maintaining blinding and allocation concealment, and ensuring accurate delivery of study medications. Establishing feasibility in these domains is critical before undertaking a fully powered multicenter RCT.

A unique strength of this pilot study is the inclusion of a post-discharge questionnaire administered to families 3 months after surgery. The questionnaire will capture participants’ perspectives on outcomes they consider most meaningful, identifying areas potentially overlooked by clinical endpoints alone. In doing so, the study seeks to achieve not only methodological rigor but also enhanced clinical and patient-centered relevance.

Finally, this work has important implications for antibiotic stewardship. PT provides broader coverage but risks promoting antimicrobial resistance and unnecessary adverse effects if not demonstrably superior to CM. Establishing whether PT offers true clinical benefit—or simply broader but avoidable exposure—is critical for balancing effectiveness with long-term safety.

To the best of our knowledge, ALPACA will be the first double-blind RCT comparing PT and CM in children with perforated appendicitis. This pilot study will determine whether a full multicenter trial is feasible and, ultimately, whether broad-spectrum therapy is justified in this common pediatric condition.

## Supporting information

S1 Appendix Ethics approved protocol. (DOCX)

S2 Appendix SPIRIT checklist. (PDF)

## References

[1] St Peter SD, Snyder CL. Operative management of appendicitis. Semin Pediatr Surg. 2016;25(4):208–11. doi: 10.1053/j.sempedsurg.2016.05.003 27521710

[2] Ruffolo C, Fiorot A, Pagura G, Antoniutti M, Massani M, Caratozzolo E, et al. Acute appendicitis: what is the gold standard of treatment? World J Gastroenterol. 2013;19(47):8799–807. doi: 10.3748/wjg.v19.i47.8799 24379603 PMC3870531

[3] Linnaus ME, Ostlie DJ. Complications in common general pediatric surgery procedures. Semin Pediatr Surg. 2016;25(6):404–11. doi: 10.1053/j.sempedsurg.2016.10.002 27989365

[4] St Peter SD, Tsao K, Spilde TL, Holcomb GW 3rd, Sharp SW, Murphy JP, et al. Single daily dosing ceftriaxone and metronidazole vs standard triple antibiotic regimen for perforated appendicitis in children: a prospective randomized trial. J Pediatr Surg. 2008;43(6):981–5. doi: 10.1016/j.jpedsurg.2008.02.018 18558169 PMC3082440

[5] Snyder KB, Hunter CJ, Buonpane CL. Perforated Appendicitis in Children: Management, Microbiology, and Antibiotic Stewardship. Paediatr Drugs. 2024;26(3):277–86. doi: 10.1007/s40272-024-00630-0 38653916

[6] Lee J, Garvey EM, Bundrant N, Hargis-Villanueva A, Kang P, Osuchukwu O, et al. IMPPACT (Intravenous Monotherapy for Postoperative Perforated Appendicitis in Children Trial): Randomized Clinical Trial of Monotherapy Versus Multi-drug Antibiotic Therapy. Ann Surg. 2021;274(3):406–10. doi: 10.1097/SLA.0000000000005006 34132703

[7] Kashtan MA, Graham DA, Melvin P, Hills-Dunlap JL, Anandalwar SP, Rangel SJ. Ceftriaxone with Metronidazole versus Piperacillin/Tazobactam in the management of complicated appendicitis in children: Results from a multicenter pediatric NSQIP analysis. J Pediatr Surg. 2022;57(10):365–72. doi: 10.1016/j.jpedsurg.2021.11.009 34876294

[8] Cramm SL, Graham DA, Feng C, Allukian M, Blakely ML, Chandler NM, et al. Use of Antipseudomonal Antibiotics is Not Associated With Lower Rates of Postoperative Drainage Procedures or More Favorable Culture Profiles in Children With Complicated Appendicitis: Results From a Multicenter Regional Research Consortium. Ann Surg. 2024;279(6):1070–6. doi: 10.1097/SLA.0000000000006152 37970676

[9] Zeineddin S, Pitt JB, Linton S, De Boer C, Hu A, Carter M, et al. Postoperative Antibiotics for Complicated Appendicitis in Children: Piperacillin/Tazobactam Versus Ceftriaxone with Metronidazole. J Pediatr Surg. 2023;58(6):1128–32. doi: 10.1016/j.jpedsurg.2023.02.027 36931937

[10] Jernigan JA, Hatfield KM, Wolford H, Nelson RE, Olubajo B, Reddy SC, et al. Multidrug-Resistant Bacterial Infections in U.S. Hospitalized Patients, 2012-2017. N Engl J Med. 2020;382(14):1309–19. doi: 10.1056/NEJMoa1914433 32242356 PMC10961699

[11] Canadian Antimicrobial Resistance Alliance. CANWARD Pathogens. Accessed 2023 February 27 http://www.can-r.com/study.php?study=canw2018&year=2018

[12] Ramirez J, Guarner F, Bustos Fernandez L, Maruy A, Sdepanian VL, Cohen H. Antibiotics as Major Disruptors of Gut Microbiota. Front Cell Infect Microbiol. 2020;10:572912. doi: 10.3389/fcimb.2020.572912 33330122 PMC7732679

[13] Antimicrobial Resistance Collaborators. Global burden of bacterial antimicrobial resistance in 2019: a systematic analysis. Lancet. 2022;399(10325):629–55. doi: 10.1016/S0140-6736(21)02724-0 35065702 PMC8841637

[14] Le Saux N, Viel-Thériault I. Shifting the antibiotic rhetoric in children from “just in case” to “disclose the risk”: Has the time come?. J Assoc Med Microbiol Infect Dis Can. 2024;9(1):6–10. doi: 10.3138/jammi-2023-12-08 38567369 PMC10984317

[15] Sarkar A, Yoo JY, Valeria Ozorio Dutra S, Morgan KH, Groer M. The Association between Early-Life Gut Microbiota and Long-Term Health and Diseases. J Clin Med. 2021;10(3):459. doi: 10.3390/jcm10030459 33504109 PMC7865818

[16] Hills RD Jr, Pontefract BA, Mishcon HR, Black CA, Sutton SC, Theberge CR. Gut Microbiome: Profound Implications for Diet and Disease. Nutrients. 2019;11(7):1613. doi: 10.3390/nu11071613 31315227 PMC6682904

[17] Gerber JS, Jackson MA, Tamma PD, Zaoutis TE, Committee on Infectious Diseases, Pediatric Infectious Diseases Society. Antibiotic Stewardship in Pediatrics. Pediatrics. 2021;147(1):e2020040295. doi: 10.1542/peds.2020-040295 33372120

[18] Patel J, Briatico D, Flageole H, Khan S, Chui L, Cran J, et al. Post-operative antibiotics for children with perforated appendicitis: exploring variability in treatment. In: Minneapolis, Minnesota, 2023.

[19] Joyce EL, Kane-Gill SL, Priyanka P, Fuhrman DY, Kellum JA. Piperacillin/Tazobactam and Antibiotic-Associated Acute Kidney Injury in Critically Ill Children. J Am Soc Nephrol. 2019;30(11):2243–51. doi: 10.1681/ASN.2018121223 31501354 PMC6830781

[20] MacIntyre IM. Prescribing medicines for patients with renal impairment. Medicine. 2024;52(1):31–5. doi: 10.1016/j.mpmed.2023.10.009

[21] Stringer MD. Anatomy of the Infant and Child. Pediatric Surgery. Springer Berlin Heidelberg. 2020:83–101. doi: 10.1007/978-3-662-43588-5_5

[22] Hall NJ, Sherratt FC, Eaton S, Reading I, Walker E, Chorozoglou M, et al. Conservative treatment for uncomplicated appendicitis in children: the CONTRACT feasibility study, including feasibility RCT. Health Technol Assess. 2021;25(10):1–192. doi: 10.3310/hta25100 33630732 PMC7958256

[23] Surgical Site Infection Event (SSI). National Healthcare Safety Network. https://www.cdc.gov/nhsn/pdfs/pscmanual/9pscssicurrent.pdf. Accessed 2023 March 22.

[24] Whitehead AL, Julious SA, Cooper CL, Campbell MJ. Estimating the sample size for a pilot randomised trial to minimise the overall trial sample size for the external pilot and main trial for a continuous outcome variable. Stat Methods Med Res. 2016;25(3):1057–73. doi: 10.1177/0962280215588241 26092476 PMC4876429

[25] Hertzog MA. Considerations in determining sample size for pilot studies. Res Nurs Health. 2008;31(2):180–91. doi: 10.1002/nur.20247 18183564

[26] Thabane L, Ma J, Chu R, Cheng J, Ismaila A, Rios LP, et al. A tutorial on pilot studies: the what, why and how. BMC Med Res Methodol. 2010;10:1. doi: 10.1186/1471-2288-10-1 20053272 PMC2824145

[27] Harris PA, Taylor R, Minor BL, Elliott V, Fernandez M, O’Neal L, et al. The REDCap consortium: Building an international community of software platform partners. J Biomed Inform. 2019;95:103208. doi: 10.1016/j.jbi.2019.103208 31078660 PMC7254481

[28] Eldridge SM, Chan CL, Campbell MJ, Bond CM, Hopewell S, Thabane L, et al. CONSORT 2010 statement: extension to randomised pilot and feasibility trials. BMJ. 2016;355:i5239. doi: 10.1136/bmj.i5239 27777223 PMC5076380

[29] Burns PB, Rohrich RJ, Chung KC. The levels of evidence and their role in evidence-based medicine. Plast Reconstr Surg. 2011;128(1):305–10. doi: 10.1097/PRS.0b013e318219c171 21701348 PMC3124652

[30] Karanicolas PJ, Farrokhyar F, Bhandari M. Practical tips for surgical research: blinding: who, what, when, why, how?. Can J Surg. 2010;53(5):345–8. 20858381 PMC2947122

[31] Rangel SJ, Kelsey J, Henry MCW, Moss RL. Critical analysis of clinical research reporting in pediatric surgery: justifying the need for a new standard. J Pediatr Surg. 2003;38(12):1739–43. doi: 10.1016/j.jpedsurg.2003.08.033 14666456

[32] Allin B, Aveyard N, Campion-Smith T, Floyd E, Kimpton J, Swarbrick K, et al. What Evidence Underlies Clinical Practice in Paediatric Surgery? A Systematic Review Assessing Choice of Study Design. PLoS One. 2016;11(3):e0150864. doi: 10.1371/journal.pone.0150864 26959824 PMC4784961

[33] Kern SE. Challenges in conducting clinical trials in children: approaches for improving performance. Expert Rev Clin Pharmacol. 2009;2(6):609–17. doi: 10.1586/ecp.09.40 20228942 PMC2835973

[34] Institute of Medicine (US) Committee on Clinical Research Involving Children, Field MJ, Behrman RE. Ethical conduct of clinical research involving children. Washington (DC): National Academies Press (US). 2004. 20669469

