# Supplementary material for: Assessing the Longitudinal outcomes of Piperacillin/tazobactam versus ceftriAxone and metronidazole for Children with perforated Appendicitis (ALPACA): A protocol for a pilot randomized controlled trial
Source: PLoS One. 2025 Nov 7;20(11):e0335991. doi: 10.1371/journal.pone.0335991 (PMC12594383; doi:10.1371/journal.pone.0335991)
Supplement: S1 Appendix — (DOCX) [file pone.0335991.s001.docx]

**Assessing the Longitudinal outcomes of Piperacillin/tazobactam versus
ceftriAxone and metronidazole for Children with perforated Appendicitis (ALPACA):
A randomized controlled trial**

Trial protocol version 18

**INVESTIGATORS**

**Principal Investigator**

Michael Livingston, MD, MSc, FRCSC

Assistant Professor of Surgery

Division of Pediatric Surgery

McMaster University

1200 Main Street West

Hamilton, Ontario, L8N-3Z5

Phone: 905-521-2100 ext.75231

Email: livingm@mcmaster.ca

**Co-Investigators**

Nadia Safa, MD, MSc, FRCSC

Pediatric General Surgery Fellow

Division of Pediatric Surgery, McMaster University

Email: nadia.safa@medportal.ca

Helene Flageole, MD, MSc, FRCSC, FACS

Professor of Surgery

Division of Pediatric Surgery, McMaster University

Email: flageol@mcmaster.ca

Sarah Khan, MD, FRCPC, DTMH

Associate Professor of Pediatrics

Division of Infectious Disease, McMaster University

Email: khan259@mcmaster.ca

Jeffery Pernica, MD, MSc, FRCPC, DTMH

Associate Professor of Pediatrics

Division of Infectious Disease, McMaster University

Email: pernica@mcmaster.ca

Mohamed Eltorki, MD, FRCPC

Associate Professor of Pediatrics

Division of Emergency Medicine, University of Calgary

Email: mmeltork@ucalgary.ca

Eyal Cohen, MD, MSc, FRCPC

Pediatrician, SickKids, University of Toronto

Email: eyal.cohen@sickkids.ca

**Research Coordinator**

Daniel Briatico, MSc

McMaster Pediatric Surgery Research Collaborative

1200 Main Street West

Hamilton, Ontario, L8N-3Z5

Phone: 905-929-9433

Email: briaticd@mcmaster.ca

TABLE OF CONTENTS

[1.0 PURPOSE AND BACKGROUND 5](#_Toc166750636)

[1.1 Background 5](#_Toc166750637)

[1.2 Literature Review 5](#_Toc166750638)

[1.3 Rationale 6](#_Toc166750639)

[2.0 OBJECTIVES 6](#_Toc166750640)

[3.0 DESIGN METHODS 6](#_Toc166750641)

[3.1 Study Setting 6](#_Toc166750642)

[3.2 Population 7](#_Toc166750643)

[3.3 Interventions 7](#_Toc166750644)

[3.4 Usual Practice 7](#_Toc166750645)

[3.5 Outcomes 8](#_Toc166750646)

[3.6 Sample Size for Pilot Study 9](#_Toc166750647)

[3.7 Sample Size for Future Multicenter Trial 9](#_Toc166750648)

[3.8 Recruitment 9](#_Toc166750649)

[3.9 Randomization 10](#_Toc166750650)

[3.10 Concealment Mechanism 10](#_Toc166750651)

[3.11 Blinding 10](#_Toc166750652)

[3.12 Follow-up 10](#_Toc166750653)

[4.0 ANALYSIS PLAN 11](#_Toc166750654)

[4.1 Data Collection 11](#_Toc166750655)

[4.2 Data Management 11](#_Toc166750656)

[4.3 Statistical Methods 11](#_Toc166750657)

[4.4 Data Monitoring 11](#_Toc166750658)

[4.5 Serious Adverse Events 12](#_Toc166750659)

[4.6 Data Safety Monitoring Board 12](#_Toc166750660)

[5.0 PROPOSED TIMELINE 12](#_Toc166750661)

[5.1 Milestones 12](#_Toc166750662)

[5.2 Dissemination 12](#_Toc166750663)

[6.0 MISCELLANEOUS 12](#_Toc166750664)

[6.1 Confidentiality 12](#_Toc166750665)

[6.2 Access to Data 12](#_Toc166750666)

[6.3 Ancillary and Post-Trial Care 13](#_Toc166750667)

[References 14](#_Toc166750668)

[Appendix 1: ALPACA Study Summary 16](#_Toc166750669)

[Appendix 2: Surgical Site Infection (SSI) Definition 17](#_Toc166750670)

# **1.0 PURPOSE AND BACKGROUND**

## **1.1 Background**

Acute appendicitis is the most common indication for emergency surgery in children.^1^ The management of this condition is pre-operative intravenous (IV) antibiotics followed by urgent laparoscopic appendectomy. If the appendix is found to be perforated at the time of surgery, then patients need to stay in hospital for post-operative IV antibiotics. Patients who do not respond to antibiotic therapy may experience prolonged length of stay, need for additional procedures (such as percutaneous drain insertion by Interventional Radiology), or other complications.^2^ This represents significant morbidity for patients and their families.

McMaster Children’s Hospital treats hundreds of children with acute appendicitis each year. Almost every week, one of these patients is found to have perforated appendicitis and needs to stay in hospital for IV antibiotics. Even though this is a common problem, we still do not know for sure which type of post-operative antibiotics is best.

## **1.2 Literature Review**

Children with perforated appendicitis were previously treated with post-operative ampicillin, gentamicin, and metronidazole (also known as “triple therapy”). In 2008, a randomized controlled trial (RCT) showed that triple therapy is non-inferior to ceftriaxone and metronidazole (CM) in terms of intra-abdominal abscess formation and wound infection.^3^ CM is also less expensive and has a simplified dosing regimen. As such, post-operative CM became the standard of care for perforated appendicitis at most children’s hospitals.

In 2021, an open-label RCT suggested that piperacillin/tazobactam (PT) is more effective than CM for children with perforated appendicitis.^4^ Patients randomized to PT had a reduced rate of intra-abdominal abscess formation compared to those treated with CM (odds ratio (OR) 4.80, p=0.002). Unfortunately, this trial was not blinded and there was no allocation concealment. There was also possible ascertainment bias, since the primary outcome was intra-abdominal abscess on post-operative imaging. The decision to obtain imaging was based on the judgement of the treating physician, who was not blinded to the type of antibiotics that each patient was receiving.

In contrast, a multicenter, observational study reported no difference in the rate of intra-abdominal abscess formation among patients treated with PT compared to CM.^5^ The conflicting results of these two studies add further uncertainty regarding the choice of antibiotics for these patients. Furthermore, these two studies were conducted in the United States and the findings may not be generalizable to other settings. There have been no comparative studies in Canadian patients, who may have gut flora with different microbial sensitivities and antibiotic resistance patterns.^6,7^

Another factor that should be considered regarding the use of PT versus CM in children with perforated appendicitis is antibiotic stewardship.^8^ PT is a broad-spectrum antibiotic with increased effectiveness against *Pseudomonas aeruginosa* and resistant *Escherichia coli*. As a result, this medication is often reserved for patients with confirmed *Pseudomonas* infection, oncology patients with febrile neutropenia, or those who are critically ill and admitted to the intensive care unit. Using PT for a routine, community-acquired infection like perforated appendicitis may result in unnecessary exposure to broad-spectrum antibiotics, which appears to be associated with long-term harms.^9-11^

##

## **1.3 Rationale**

At McMaster Children’s Hospital, we conducted a multidisciplinary quality assurance project that documented significant variation in the type of post-operative antibiotics used among children with perforated appendicitis (n=71).^12^ We found that some patients received CM alone (n=32), others received PT (n=23), and others still were treated with CM initially and “escalated” to PT if their symptoms did not improve (n=15). Compared to patients treated with CM only (n=32), those escalated to PT (n=15) had worse intra-operative findings at baseline, as well as increased need for post-operative ultrasound (67% vs 34%, p=0.038), parenteral nutrition (33% vs 3%, p=0.004), peripherally inserted central catheter (PICC) insertion (47% vs 3%, p<0.001), and increased length of stay (10.7 vs 5.1 days, p<0.001). There were 3 cases of *Clostridium difficile* infection (4%), all in patients who received CM.

In short, there is clinical equipoise regarding the use of PT or CM in children with perforated appendicitis. There is uncertainty in the published literature and significant variation in practice at our center. A single RCT with substantial risk of bias reported evidence of benefit with the use of PT, while a multicenter, observational study showed no difference. Given the potential harm and unnecessary exposure to broad-spectrum antibiotics, this study will address this important knowledge gap.

# **2.0 OBJECTIVES**

The Assessing Longitudinal outcomes of Piperacillin/tazobacatAm versus ceftriaxone/metronidazole for Children with perforated Appendicitis (ALPACA) trial is an internal pilot study for a multicenter, blinded RCT using two parallel groups.

The research question for the multicenter trial is: “Among children less than 18 years of age who undergo laparoscopic appendectomy for perforated appendicitis, is post-operative piperacillin/tazobactam superior to ceftriaxone and metronidazole in terms of length of stay in hospital?”

The purpose of conducting an internal pilot study is to ensure that the multicenter trial is feasible. The challenges of conducting such a trial include: (1) the need to identify and enroll participants prior to emergency surgery; (2) the need to efficiently obtain informed consent and assent in pediatric patients; (3) the logistical difficulties of randomizing participants after an emergency procedure; and (4) ensuring participants receive blinded study treatments (i.e., post-operative antibiotics) in a timely fashion.

# **3.0 DESIGN METHODS**

## **3.1 Study Setting**

The internal pilot study will be conducted at McMaster Children’s Hospital, a pediatric tertiary care center located in Hamilton, Ontario, Canada. See Appendix 1 for a study summary

## **3.2 Population**

***Inclusion Criteria:***

- Age less than 18 years
- Laparoscopic appendectomy
- Perforated appendicitis confirmed intra-operatively (i.e., visible hole in appendix, fecalith found in peritoneal cavity, intra-abdominal abscess, and/or purulent fluid in peritoneal cavity)

***Exclusion Criteria:***

- Non-operative treatment (e.g., due to abscess)
- Interval laparoscopic appendectomy
- Conversion to open procedure
- Non-perforated appendicitis
- Confirmed or suspected allergy to penicillins or cephalosporins
- Renal impairment
- Weight less than 10 kilograms

## **3.3 Interventions**

Participants will be randomized to receive one of the following post-operative antibiotic regimens:

1. Piperacillin/tazobactam 100 mg/kg IV q8h (to a maximum of 4.5 g IV q8h) and normal saline once daily:
2. Ceftriaxone 50 mg/kg IV once daily (to a maximum of 2 g IV daily) and metronidazole 10 mg/kg IV q8h (to a maximum of 500 mg IV q8h):

All participants will receive one study treatment every 8 hours (either metronidazole or piperacillin/tazobactam) and one study treatment every 24 hours (either ceftriaxone or normal saline).

## **3.4 Usual Practice**

At McMaster Children’s Hospital, all children who undergo laparoscopic appendectomy are treated by pediatric general surgeons with fellowship training in Pediatric Surgery. Patients found to have perforated appendicitis are treated with CM or PT in hospital post-operatively. Pain is controlled with oral Tylenol, IV Ketororolac (or oral ibuprofen), and IV morphine as needed. Maintenance IV fluids are gradually weaned as an oral diet is introduced.

Patients who are unable to tolerate an oral diet for 7 days (which consists of the total days before and after surgery) are often considered for PICC insertion and parenteral nutrition. Ultimately, however, decisions about timing of PICC insertion and starting parenteral nutrition are left to clinician judgement. The duration of IV antibiotics at our institution is based on clinical criteria. These include: (1) resolution of pain and localized tenderness; (2) resolution of fevers (i.e., any documented temperature greater than 38.0 degrees); (3) resolution of diarrhea; and (4) ability to tolerate an oral diet. If symptoms resolve within one week of surgery, patients are discharged home with a course of oral antibiotics, which is usually amoxicillin and clavulanic acid (i.e., Clavulin®).

Patients who continue to have symptoms up to one week after surgery undergo abdominal ultrasound to assess for the presence of a phlegmon (i.e., inflamed soft tissue) or drainable abscess (i.e., purulent fluid). Patients with a large abscess (i.e., greater than 5 cm in maximal dimension) are often considered for percutaneous drain insertion by Interventional Radiology. This procedure requires a general anesthetic in children and the drain is left in place for a few days to allow the abscess to evacuate completely. These patients remain in hospital on IV antibiotics until the drain is removed and symptoms resolve.

When patients are discharged home, they are advised to contact our office or return to the emergency department if they have fevers, signs of a surgical site infection (SSI) (i.e., erythema, drainage, worsening incisional pain, edema, etc.), persistent diarrhea (which may be a sign of an intra-abdominal abscess or *Clostridium difficile* infection), abdominal pain, or inability to tolerate an oral diet. Some patients who return to the emergency department require readmission to hospital for additional IV antibiotics and possible percutaneous drain insertion.

## **3.5 Outcomes**

***Feasibility Outcomes***

- Recruitment rate (i.e., number of participants randomized per month)
- Consent rate (i.e., number of participants who consent to participate divided by those who are approached for consent)
- Rate of protocol violations (i.e., number of participants who do not receive study treatments within 8 hours of surgery, miss a scheduled study treatment, and/or experience treatment crossover divided by those randomized)
- Rate of loss to follow-up (i.e., number of participants who cannot be contacted by phone 3 months after discharge from hospital divided by those randomized)
- Cost per participant randomized (i.e., total cost of internal pilot study in Canadian dollars divided by the number of participants randomized)

Our criteria for feasibility are recruitment rate≥1 new participant per month, consent rate>30%, rate of protocol violations<20%, rate of loss to follow-up<10%, and cost per participant randomized<3000 Canadian dollars. A similar RCT of children with appendicitis reported a consent rate of 50%.^13^ This increased from 38% to 72% throughout the course of the study with focused training of clinical and research personnel.

***Primary Outcome***

The primary outcome for the multicenter RCT is length of stay in hospital. Patients who respond to post-operative antibiotics remain in hospital for approximately 5 days. Patients who experience prolonged stays in hospital, however, often do so because of persistent infectious symptoms (i.e., fever, abdominal pain, diarrhea, and/or poor oral intake) with possible need for percutaneous drain insertion. Length of stay for these patients is typically one to two weeks (or longer).

***Secondary Outcomes***

We will also collect data for a variety of secondary outcomes. These are likely to be affected by the type of antibiotic therapy and have been used in other trials of children with perforated appendicitis. Secondary outcomes include:

- Post-operative ultrasound or computed tomography (CT)
- Deep or organ-space SSI (see Appendix 2)
- Percutaneous drain insertion
- *Clostridium difficile* infection (confirmed with stool sample and requiring treatment)
- Parenteral nutrition
- PICC insertion
- Return to the emergency department within 30 days of surgery
- Readmission to hospital within 30 days of surgery

***Patient-reported Outcomes***

The Research Coordinator for the ALPACA Study will call all families 3 months after surgery to ensure that they have not experienced any additional complications related to perforated appendicitis. Parents or patients capable of giving consent will also complete a questionnaire by phone regarding patient satisfaction and rank the relative importance of the primary and secondary outcomes (see Appendix 3 and Appendix 4).

## **3.6 Sample Size for Pilot Study**

The sample size of the internal pilot study will be 16 participants (i.e., 8 per treatment arm). This number is approximately 10% of the total sample size for the multicenter randomized controlled trial and should be sufficient to assess feasibility and cost.^13-16^

## **3.7 Sample Size for Future Multicenter Trial**

The sample size for the multicenter RCT is 168 participants (i.e., 84 per treatment arm). In our recent quality improvement project, the length of stay of participants treated with PT only (n=23) was 5.9 days (standard deviation 2.9 days) whereas those who received CM first (n=32) was 6.9 days (standard deviation 4.2 days).^12^ The standard deviation for the entire sample (n=71) was 3.8 days. If the alpha level is 0.05 and power is 90%, and the study is powered to detect a difference in length of stay of at least 2 days, then this trial would require a total of 152 participants. Assuming a dropout rate of approximately 10%, then we plan to increase the total sample size to 168 participants.

## **3.8 Recruitment**

Participants for the ALPACA Study will be identified by the attending pediatric surgeon or Pediatric Surgery fellow. After consent is obtained for surgery (i.e., “laparoscopic possibly open appendectomy”), the patient and family will be asked if they are agreeable to learn more about this study. If so, they will be approached by a member of the research team for possible study enrollment. Informed consent will be obtained from the parent or legal guardian. Participants who are not consented before surgery will not be enrolled. Assent will obtained from the patient whenever possible.

Patients who decline to participate will receive usual clinical care. This includes laparoscopic appendectomy followed by discharge home if the appendix is not perforated at the time of surgery. If the appendix is found to be perforated, patients will receive PT or CM post-operatively (with the type of antibiotics left to the discretion of the attending pediatric surgeon).

Patients and families who consent to participate in the ALPACA Study will also proceed with surgery as per usual clinical care. If the appendix is found to be perforated at the time of surgery, they will be randomized to receive either PT or CM post-operatively.

## **3.9 Randomization**

Participants will be randomized to treatment groups using a computer-generated randomization list. This will consist of random blocks of multiple sizes ranging 2 to 6 individuals, created by a senior biostatistician. Participants will be randomized according to a 1:1 parallel allocation, with an equal chance of being allocated PT or CM.

## **3.10 Concealment Mechanism**

The randomization scheme will be housed in an online web-based randomization system within Research Electronic Data Capture (REDCap).^17^ Participants will be randomized by the research pharmacy at McMaster Children’s Hospital once they have been enrolled in the study, assuring allocation concealment from study investigators and the clinical team. The inpatient pharmacist will possess a study key to determine which study arm the patient is assigned to. The assigned treatments will be crosschecked against the master linkage key at the end of the study.

## **3.11 Blinding**

Participants, families, bedside nurses, attending pediatric surgeons, Pediatric Surgery fellows, surgical trainees, and outcome assessors will remain blinded throughout the trial. The only reason for emergency unblinding is if a participant develops signs of moderate to severe allergic reaction (i.e., hives, anaphylaxis, etc.). This will allow the clinical team to determine if the reaction was possibly due to a penicillin (i.e., PT) or cephalosporin (i.e., ceftriaxone). Allergic reactions to IV metronidazole are rare. After unblinding, the patient’s IV antibiotics will be changed to an alternative regimen. This is typically ciprofloxacin and metronidazole, but the decision will be left to the clinical team.

## **3.12 Follow-up**

All children who undergo laparoscopic appendectomy for perforated appendicitis at McMaster Children’s Hospital are routinely followed by two trained National Surgical Quality Improvement Program (NSQIP) data abstractors. Outcomes measured for all patients who undergo laparoscopic appendectomy include:

- Length of stay
- Parenteral nutrition
- Deep or organ-space SSI (see definition in Appendix 2)
- Sepsis
- Admission to intensive care unit
- Return to the operating room
- Return to the emergency department within 30 days of discharge
- Readmission to hospital

There should be no loss to follow-up while study participants remain in hospital. After discharge, the two NSQIP abstractors routinely review the electronic medical record up to 30 days post-operatively. This is to assess for return to the emergency department or readmission to hospital. There is a possibility that participants could develop a SSI (or other complication) and present to the emergency department at a different hospital. To capture this, our Research Coordinator will follow-up via telephone 3 months after discharge from our centre (see Appendix 3).

# **4.0 ANALYSIS PLAN**

## **4.1 Data Collection**

The Research Coordinator will be responsible for storing signed consent forms and entering baseline data. Feasibility outcomes will also be assessed by the Research Coordinator. The NSQIP Data Abstractors will be responsible for assessing and recording the primary and secondary outcomes.

## **4.2 Data Management**

All data for this study will be collected and entered into a secure, online REDCap database, designed specifically for the purpose of this study. All data entered into the REDCap database will be de-identified. All patient identifiers will be replaced with a study identification number. All study files containing patient identifiers, including the master list linking participant medical record number (MRN) to study identification number, will be stored in password-protected excel files on the secure drive for the Department of Surgery at McMaster University. This drive is protected by the McMaster University Firewall and only research staff directly involved in the conduct of this trial will have access to these files. Paper consent forms will be stored in a locked cabinet in the secure office area for the Department of Surgery. Only research staff directly involved in the conduct of this trial will have access to this cabinet.

Data checks will be completed throughout the trial to ensure the accuracy of all data entered into the REDCap database. These checks will be completed after 5 participants are randomized, with an additional check after the internal pilot study is completed.

## **4.3 Statistical Methods**

Data will be stored in REDCap and analyzed in the Statistical Package for the Social Sciences (SPSS). Categorical data will be presented as frequency (n) and proportion (%), and analyzed using Chi-squared tests. Continuous data will be presented as mean and standard deviation (SD), and analyzed using independent t-tests. P-values less than 0.05 will be considered statistically significant. Missing data will not be imputed and those cases will be excluded.

During the internal pilot study, hypothesis testing will not be conducted on any of the primary or secondary outcomes, since there is insufficient statistical power to support these analyses. Cost per participant randomized will estimated in Canadian dollars. This will include the personnel costs associated with consent, randomization, outcome assessment, and pharmacy. These data will eventually be used to estimate the length of time needed to recruit participants in the full-scale trial.

## **4.4 Data Monitoring**

This study will employ a Steering Committee. The Steering Committee members will be responsible for overseeing the conduct of the trial. The Steering Committee will meet monthly, and will involve representatives from Pediatric Surgery and Pediatric Infectious Disease.

## **4.5 Serious Adverse Events**

A serious adverse event (SAE) is defined as any untoward medical occurrence that results in death, is life-threatening, requires inpatient hospitalization or prolongation of existing hospitalization, results in persistent or significant disability/incapacity, or is a congenital anomaly/birth defect. SAE reporting will adhere to the guidelines set out by HiREB, which requires reporting of all SAE that are unexpected and related to study treatments (or possibly related). If an unexpected SAE occurs, the Principal Investigator will notify the Data Safety Monitoring Board (DSMB) within 48 hours. The only SAE that will be reviewed by the DSMB are allergic reactions possibly related to study medications. The DSMB will also review any cases of mortality within 30 days of surgery.

## **4.6 Data Safety Monitoring Board**

This study will utilize a DSMB. The DSMB will consist of three healthcare professionals independent of the Steering Committee who will monitor patient safety during the conduct of this study. Their role is to review all SAE that are unexpected and determine if they are related or possibly related to study treatments. The DSMB will submit a summary report to the Steering Committee and HiREB. Based on these reports, the DSMB will recommend either continuing or discontinuing the trial due to harm.

# **5.0 PROPOSED TIMELINE**

## **5.1 Milestones**

Our milestones include obtaining ethics approval from the Hamilton Integrated Research Ethics Board (HiREB) by 6 months and enrolling participants by one year. At McMaster Children’s Hospital, approximately 60 pediatric patients undergo laparoscopic appendectomy for perforated appendicitis each year. As such, it is estimated that recruitment for the pilot study will require at least one year of enrollment. This is based on an estimated recruitment rate of 2-3 participants randomized per month and an estimated consent rate of 50%.^13^

## **5.2 Dissemination**

The results of the internal pilot study will be published in a peer-reviewed journal and presented at relevant academic meetings. These include the annual meeting for the Canadian Association of Pediatric Surgeons and the Canadian Pediatric Society.

# **6.0 MISCELLANEOUS**

## **6.1 Confidentiality**

The confidentiality of each patient’s medical information will be treated with the strictest of confidence and in accordance with both Good Clinical Practice (GCP) guidelines and the Personal Health Information Protection Act (PHIPA).^18^ Each study patient will be given a unique identification number and all case report forms will be kept in a secure filing cabinet in a locked office. We will not collect any personal identifiers that are not absolutely necessary to the success of this study. All data will be anonymized for data validation and analysis.

## **6.2 Access to Data**

Only the research staff directly involved in the conduct of the study will have access to the de-identified study data and the participant master list. All data sets will be password protected.

## **6.3 Ancillary and Post-Trial Care**

All trial data will be preserved for 15 years in keeping with GCP guidelines. Interested participants and their families can be advised of the results of the internal pilot study by email.

# **References**

1. St Peter SD, Snyder CL. Operative management of appendicitis. Semin Pediatr Surg. 2016 Aug;25(4):208-11. PMID: 27521710.
2. Linnaus ME, Ostlie DJ. Complications in common general pediatric surgery procedures. Semin Pediatr Surg. 2016 Dec;25(6):404-411. PMID: 27989365.
3. St Peter SD, Tsao K, Spilde TL, Holcomb GW 3rd, Sharp SW, Murphy JP, Snyder CL, Sharp RJ, Andrews WS, Ostlie DJ. Single daily dosing ceftriaxone and metronidazole vs standard triple antibiotic regimen for perforated appendicitis in children: a prospective randomized trial. J Pediatr Surg. 2008 Jun;43(6):981-5. PMID: 18558169.
4. Lee J, Garvey EM, Bundrant N, Hargis-Villanueva A, Kang P, Osuchukwu O, Dekonenko C, Svetanoff WJ, St Peter SD, Padilla B, Ostlie D. IMPPACT (Intravenous Monotherapy for Postoperative Perforated Appendicitis in Children Trial): Randomized Clinical Trial of Monotherapy Versus Multi-drug Antibiotic Therapy. Ann Surg. 2021 Sep 1;274(3):406-410. PMID: 34132703.
5. Kashtan MA, Graham DA, Melvin P, Hills-Dunlap JL, Anandalwar SP, Rangel SJ. Ceftriaxone with Metronidazole versus Piperacillin/Tazobactam in the management of complicated appendicitis in children: Results from a multicenter pediatric NSQIP analysis. J Pediatr Surg. 2022 Oct;57(10):365-372. PMID: 34876294.
6. Jernigan JA, Hatfield KM, Wolford H, Nelson RE, Olubajo B, Reddy SC, McCarthy N, Paul P, McDonald LC, Kallen A, Fiore A, Craig M, Baggs J. Multidrug-Resistant Bacterial Infections in U.S. Hospitalized Patients, 2012-2017. N Engl J Med. 2020;382:1309-1319. PMID: 32242356.
7. Canadian Antimicrobial Resistance Alliance. CANWARD Pathogens. http://www.can-r.com/study.php?study=canw2018&year=2018. Accessed 27 February 2023.
8. Ramirez J, Guarner F, Bustos Fernandez L, Maruy A, Sdepanian VL, Cohen H. Antibiotics as Major Disruptors of Gut Microbiota. Front Cell Infect Microbiol. 2020;10:572912. PMID: 33330122.
9. Sarkar A, Yoo JY, Valeria Ozorio Dutra S, Morgan KH, Groer M. The Association between Early-Life Gut Microbiota and Long-Term Health and Diseases. J Clin Med. 2021;10:459. PMID: 33504109.
10. Hills RD Jr, Pontefract BA, Mishcon HR, Black CA, Sutton SC, Theberge CR. Gut Microbiome: Profound Implications for Diet and Disease. Nutrients. 2019 Jul 16;11(7):1613. doi: 10.3390/nu11071613. PMID: 31315227.
11. Gerber JS, Jackson MA, Tamma PD, Zaoutis TE; COMMITTEE ON INFECTIOUS DISEASES, PEDIATRIC INFECTIOUS DISEASES SOCIETY. Antibiotic Stewardship in Pediatrics. Pediatrics. 2021 Jan;147(1):e2020040295. PMID: 33372120.
12. Patel J, Briatico D, Flageole H, Khan S, Chui L, Cran J, Livingston MH. (2023) Post-operative antibiotics for children with perforated appendicitis: exploring variability in treatment. American College of Surgeons Quality and Safety Annual Meeting, Minneapolis, Minnesota (July 7).
13. Hall NJ, Eaton S, Sherratt FC, Reading I, Walker E, Chorozoglou M, Beasant L, Wood W, Stanton M, Corbett H, Rex D, Hutchings N, Dixon E, Grist S, Crawley EM, Young B, Blazeby JM. CONservative TReatment of Appendicitis in Children: a randomised controlled feasibility Trial (CONTRACT). Arch Dis Child. 2021;106:764–73. PMID: 33441315.
14. Whitehead AL, Julious SA, Cooper CL, Campbell MJ. Estimating the sample size for a pilot randomised trial to minimise the overall trial sample size for the external pilot and main trial for a continuous outcome variable. Stat Methods Med Res. 2016;25:1057-73. PMID: 26092476.
15. Hertzog MA. Considerations in determining sample size for pilot studies. Res Nurs Health. 2008 Apr;31(2):180-91. PMID: 18183564.
16. Thabane L, Ma J, Chu R, Cheng J, Ismaila A, Rios LP, Robson R, Thabane M, Giangregorio L, Goldsmith CH. A tutorial on pilot studies: the what, why and how. BMC Med Res Methodol. 2010 Jan 6;10:1. PMID: 20053272.
17. Harris PA, Taylor R, Minor BL, Elliott V, Fernandez M, O'Neal L, McLeod L, Delacqua G, Delacqua F, Kirby J, Duda SN; REDCap Consortium. The REDCap consortium: Building an international community of software platform partners. J Biomed Inform. 2019 Jul;95:103208. PMID: 31078660.
18. ICH Harmonized Guideline. Integrated addendum to ICH E6 (R1): Guideline for good clinical practice E6 (R2). Current Step. 2016 Nov;4:1-66.
19. Surgical Site Infection Event (SSI). National Healthcare Safety Network. <https://www.cdc.gov/nhsn/pdfs/pscmanual/9pscssicurrent.pdf>. January 2023. Accessed 22 March 2023.

# **Appendix 1: ALPACA Study Summary**

| **Running title** | ALPACA Study |
| --- | --- |
| **Participants** | Children <18 years with perforated appendicitis treated with laparoscopic appendectomy |
| **Intervention** | Post-operative piperacillin/tazobactam 100 mg/kg IV q8h (to a maximum of 4.5 g IV q8h) and normal saline 100 mL once daily |
| **Control** | Post-operative ceftriaxone 50 mg/kg IV once daily (to a maximum of 2 g IV daily) and metronidazole 10 mg/kg IV q8h (to a maximum of 500 mg IV q8h) |
| **Feasibility outcomes** | Recruitment rate, consent rate, rate of protocol violations, rate of loss to follow-up, cost per patient randomized |
| **Criteria for feasibility** | Recruitment rate≥1 new participant per month, consent rate>30%, rate of protocol violations<20%, rate of loss to follow-up<10%, cost per participant randomized<3000 Canadian dollars |

**Appendix 2: Surgical Site Infection (SSI) Definition by the National Healthcare Safety Network^19^**

***Deep SSI:***

Date of event occurs within 30 or 90 days following the… operative procedure (where day 1 = the procedure date) …

**AND**

involves deep soft tissues of the incision (for example, fascial and muscle layers)

**AND**

patient has at least one of the following:

1. purulent drainage from the deep incision.
2. a deep incision that is deliberately opened or aspirated by a surgeon, physician* or physician designee or spontaneously dehisces
   **AND**
   organism(s) identified from the deep soft tissues of the incision by a culture or non-culture based microbiologic testing method which is performed for purposes of clinical diagnosis or treatment (for example, not Active Surveillance Culture/Testing) or culture or nonculture based microbiologic testing method is not performed. A culture or non-culture based test from the deep soft tissues of the incision that has a negative finding does not meet this criterion.
   **AND**
   patient has at least one of the following signs or symptoms: fever (>38°C); localized pain or tenderness.
3. an abscess or other evidence of infection involving the deep incision detected on gross anatomical exam, histopathologic exam, or imaging test.

* The term physician for the purpose of application of the NHSN SSI criteria may be interpreted to mean a surgeon, infectious disease physician, emergency physician, other physician on the case, or physician’s designee (nurse practitioner or physician’s assistant).

***Organ/Space SSI***

Date of event occurs within 30 or 90 days following the … operative procedure (where day 1 = the procedure date) …

**AND**

involves any part of the body deeper than the fascial/muscle layers that is opened or manipulated during the operative procedure

**AND**

patient has at least one of the following:

1. purulent drainage from a drain placed into the organ/space (for example, closed suction drainage system, open drain, T-tube drain, CT-guided drainage)
2. organism(s) identified from fluid or tissue in the organ/space by a culture or non-culture based microbiologic testing method which is performed for purposes of clinical diagnosis or treatment (for example, not Active Surveillance Culture/Testing
3. an abscess or other evidence of infection involving the organ/space detected on gross anatomical exam or histopathologic exam, or imaging test evidence definitive or equivocal for infection

**AND**

meets at least one criterion for a specific organ/space infection site.
